# Teacher Burnout in the Time of COVID-19: Antecedents and Psychological Consequences

**DOI:** 10.3390/ijerph20054204

**Published:** 2023-02-27

**Authors:** Anita Padmanabhanunni, Tyrone B. Pretorius

**Affiliations:** Department of Psychology, University of the Western Cape, Bellville 7530, South Africa

**Keywords:** burnout antecedents, burnout consequences, psychological well-being

## Abstract

The important, frontline role of teachers during the COVID-19 pandemic has often gone unrecognized, and attention to their mental health and well-being is often only the focus of scholarly research. The unprecedented challenges that teachers faced during the COVID-19 pandemic and the stresses and strains associated with it have severely impacted their psychological well-being. This study examined the predictors and the psychological consequences of burnout. Participants (N = 355) were schoolteachers in South Africa who completed the Perceived Vulnerability to Disease Questionnaire, the Fear of COVID-19 Scale, the Role Orientation Questionnaire, the Maslach Burnout Inventory, the Centre for Epidemiological Depression Scale, the Beck Hopelessness Scale, the Satisfaction with Life Scale, and the trait scale of the State-Trait Anxiety Inventory. The results of a multiple regression showed that fear of COVID-19, role ambiguity, and role conflict were significant predictors of emotional exhaustion and depersonalization, while perceived infectability and role ambiguity significantly predicted personal accomplishment. Gender and age also predicted emotional exhaustion and depersonalization, respectively, and age was also a significant predictor of personal accomplishment. Generally, the dimensions of burnout were significant predictors of indices of psychological well-being—namely, depression, hopelessness, anxiety, and life satisfaction—with the exception of the association between depersonalization and life satisfaction. Our results suggest that intervention efforts to reduce burnout need to provide teachers with adequate job resources to buffer against the demands and stressors associated with their work.

## 1. Introduction

The COVID-19 pandemic profoundly impacted the education sector in many countries. To curb the spread of the disease, governments around the world implemented severe restrictions, including the closure of all educational institutions. This necessitated a transition to emergency remote teaching, resulting in unprecedented shifts from typical instructional practices [1]. The digitization of the educational process substantially increased teachers’ working hours as they needed to master the use of information communication technology, implement new pedagogical practices, and guide their students in navigating an online learning environment [2]. Teachers also had to manage the shifts in educational policies and practices that occurred during the various stages of the pandemic, contend with their own fears related to COVID-19, and manage domestic responsibilities, including caring for their own children, homeschooling, and supporting elderly family members [3].

Prior research has confirmed that teaching is a particularly stressful occupation and is associated with high rates of burnout and teacher attrition. In the United States, 46% of teachers have reported high levels of daily stress, a rate that was only matched by nurses [4]. Sources of stress for teachers include the demands of their jobs, lack of resources, lack of support from school leadership, disengaged students, discipline problems, and difficult relationships with parents [4,5]. Teacher stress has also been negatively related to job performance [6] and psychological well-being [7] and positively related to absenteeism [8] and turnover intention [7]. It has also been demonstrated that teachers’ stress is negatively related to students’ social adjustment and academic performance [9].

For teachers, the stressors associated with their profession, along with the demands of the pandemic, can significantly impact their mental health and lead to burnout. Job burnout is defined as a psychological syndrome that results from exposure to chronic job-related stress [10]. According to Maslach’s [11] multidimensional theory of burnout, the core features of burnout are an overwhelming experience of exhaustion, a sense of cynicism and detachment from the job, appraisals of ineffectiveness, and a lack of a sense of accomplishment in the work environment. The construct of burnout consists of three dimensions: emotional exhaustion (feelings of being emotionally drained); depersonalization (an indifferent attitude toward work); and reduced personal accomplishment (negatively evaluating work-related achievements) [11]. Various studies conducted during the pandemic have highlighted increased levels of burnout among school teachers. For example, a study of German in-service teachers [2] reported elevated levels of burnout pre- and post-pandemic, particularly with regard to depersonalization and lack of personal accomplishment. Similarly, in a Spanish study, Sánchez-Pujalte and colleagues [12] found high levels of teacher emotional exhaustion during the pandemic. Female teachers were more affected by burnout compared to male teachers, while older and more experienced teachers experienced lower levels of distress. A study of Canadian teachers [13] also found increased emotional exhaustion and cynicism among school teachers. However, teachers in the sample reported a heightened sense of accomplishment as the pandemic progressed, a phenomenon attributed to experiencing a greater sense of efficacy in the management of student behavior online. Burnout can significantly impact teaching effectiveness, teachers’ interactions with students and parents, teacher motivation, and teachers’ ability to support their students and peers. Burnout has been found to correlate with job satisfaction [14], absenteeism [15], intention to quit [3], and job performance [15]. Several studies have also reported on the negative impact of burnout on indices of psychological well-being, including depression [16,17,18], anxiety [16,18], hopelessness [19,20], and suicide ideation [21,22]. Studies investigating predictors of teacher burnout have identified gender, age, self-efficacy, and institutional support as salient factors [23].

The current study uses the job demands–resources (JDR) model [24] as a lens for examining the predictors and psychological consequences of burnout among schoolteachers. The JDR model is a transactional model that has been used to understand and explain stress and burnout among schoolteachers [24]. It attributes stress and resulting burnout to a mismatch between the demands of the job and the personal (e.g., sense of self-efficacy) and organizational resources (e.g., support from managers) available to an individual [24]. Job demands refer to the features of the job that require sustained cognitive and emotional effort; job resources refer to the internal and external features of the job that facilitate the achievement of work-related tasks and reduce physical and psychological demands while also promoting personal learning and growth [24]. Job demands have the potential to contribute to role conflict and role ambiguity and can increase teachers’ vulnerability to stress and adverse mental health outcomes [2]. Role conflict occurs when there are conflicting expectations in the workplace, while role ambiguity refers to uncertainty regarding the key requirements of a job and how to accomplish them [25]. Existing studies (e.g., [25]) have reported a significant association between role conflict and role ambiguity and burnout. The JDR model is sensitive to the changes in demands and resources that may occur over time. For example, some teachers who were able to negotiate the initial demands of COVID-19-related prevention measures may have found their resources depleted during subsequent waves of the pandemic, leading to emotional exhaustion and burnout.

The current study was conducted in South Africa after its initial move to online and digital education pandemic prevention measures entailed the closure of all educational institutions and the transition to emergency remote learning and teaching. However, the socioeconomic circumstances of many learners meant that many of them had no access to either technology or Wi-Fi [26]. For this reason, the government instated rotational teaching where students would attend traditional schooling on a rotational basis. A significant proportion of South African schools are located in rural or disadvantaged community settings where access to resources and facilities (e.g., running water and sanitation) are limited. In addition, overcrowded classrooms make it difficult for teachers to implement physical distancing requirements.

One study [27] examined the role of demographic variables (gender and age), COVID-19-related variables (perceived vulnerability to disease and fear of COVID-19), and role stress (role conflict and ambiguity) as potential predictors of burnout. In addition, it also examined dimensions of burnout as potential predictors of certain indices of psychological well-being—namely, depression, hopelessness, anxiety, and life satisfaction. The categorization of the variables as antecedents and consequences of burnout is presented in Table 1.

## 2. Materials and Methods

### 2.1. Participants

Participants were school teachers (*N* = 355) from across South Africa. The majority resided in the Western Cape Province (82.3%), were women (76.6%), worked in an urban area (61.7%), and taught at the primary school level (61.1%). The mean age of the sample was 41.89 (SD = 12.42), and the mean number of years in the teaching profession was 15.7 (SD = 11.75). Our sample compared favorably with population data as reported in an international survey of teaching and learning, and we found no significant differences between the demographics in our sample and those of the international survey. This international survey [28] found that 60% of teachers in South Africa are women (*χ*^2^ = 0.06, *p* > 0.05), with a mean age of 43 (*t* = 1.68, *p* > 0.05), and a mean working experience of 15 years (*t* = 1.11, *p* > 0.05). In terms of COVID-19 status, 44.5% indicated that they had not contracted the virus. A smaller proportion of teachers either suspected that they had had COVID-19 (6.8%) but had not tested for the disease, or suspected that they had the virus and confirmed this through testing (16.6%). The survey took on average 20 minutes to complete and was only available in English. However, English is a compulsory language at schooling level, and also the medium of instruction at higher education institutions where teachers receive their training.

### 2.2. Measures

In addition to a brief demographic survey, participants completed the following questionnaires: the Perceived Vulnerability to Disease Questionnaire (PVD-Q) [29]; the Fear of COVID-19 Scale (FCV-19S) [30]; the Role Orientation Questionnaire [31]; the Maslach Burnout Inventory (MBI) [11]; the Centre for Epidemiological Depression Scale (CES-D) [32]; the Beck Hopelessness Scale (BHS) [33]; the Satisfaction with Life Scale (SWLS) [34]; and the trait scale of the State-Trait Anxiety Inventory (STAI-T) [35].

The PVD-Q assesses beliefs about personal vulnerability to infectious diseases. Duncan and colleagues [29], in a comprehensive psychometric analysis, demonstrated that the scale consists of two conceptually distinct subscales—namely, Germ Aversion (GA) and Perceived Infectability (PI). Furthermore, these two subscales appear to have different relationships with other variables. The GA subscale (eight items) assesses emotional discomfort in circumstances associated with a high potential for disease transmission. An example item from the GA subscale is: “I prefer to wash my hands pretty soon after shaking someone’s hand.” The PI subscale (seven items) assesses beliefs regarding the person’s susceptibility to infectious diseases. An example item from the PI subscale is: “If an illness is going around, I will get it.” Responses to the 15 items are scored on a 7-point Likert scale that ranges from strongly disagree (1) to strongly agree (7). Scores on the GA subscale range between 8 and 56, while on the PI subscale they range between 7 and 49. Higher scores on the GA subscale reflect a higher level of discomfort with the potential for disease transmission. Higher scores on the PI reflect higher levels of perceived infectability. The authors of the scales provided evidence of the discriminant and convergent validity of the two subscales and reported estimates of internal consistency of 0.87 and 0.74 for the PI and GA subscales, respectively [29]. However, other studies have generally reported moderate reliability coefficients for the GA subscale (e.g., α = 0.59 [36] and α = 0.56 [37]).

The FCV-19S is a 7-item scale that measures emotional fear reactions toward the pandemic. Responses are scored on a 5-point Likert scale that ranges from 1 (strongly disagree) to 5 (strongly agree). The total score ranges between 7 and 35, with a higher score reflecting a higher level of fear of COVID-19. The initial validation study [30] provided evidence of concurrent validity and satisfactory internal consistency reliability (α = 0.82). The scale has been used in a variety of contexts (e.g., Israel, Mozambique, New Zealand, and Brazil) and, generally, alpha coefficients > 0.80 have been reported [38,39,40]. Two South African studies have also confirmed the reliability and the unidimensional nature of the scale [41,42].

The Role Orientation Questionnaire assesses two dimensions related to perceptions of role stress—namely, role conflict (RC) and role ambiguity (RA). The RC (eight items) reflects the degree of dissonance experienced with regard to role expectations. An example item from RC is: “I have to work on unnecessary things.” RA (six items) reflects lack of clarity regarding role expectations. An example item from RA is: “I know exactly what is expected of me.” Responses are scored on a 6-point Likert-type scale that ranges from definitely not true of my job (1) to definitely true of my job (6). Scores on the RC subscale range between 8 and 48, while on the RA subscale they range between 6 and 36. High scores on the two scales indicate higher levels of role conflict and ambiguity. The original study [31] reported internal consistency coefficients of 0.87 and 0.82 for role ambiguity and role conflict, respectively. More recent studies have also reported satisfactory reliability coefficients (e.g., RC = 0.81, RA = 0.85 [43]; RC = 0.92, RA = 0.91 [44]).

The MBI is one of the most widely used measures of burnout. It consists of 22 items that assess three dimensions of burnout: emotional exhaustion (EE: nine items), depersonalization (DP: five items), and personal accomplishment (PA: eight items). The EE subscale is regarded as the core component of burnout and describes feelings of tiredness, fatigue, and drained emotional energy resulting from work. The DP subscale describes negative and indifferent feelings toward students and colleagues, including feelings of callousness and cynicism. The PA scale refers to sense of accomplishment and effectiveness in relation to work. Scores on the EE subscale range between 0 and 54, on the DP subscale between 0 and 30, and on the PA subscale between 0 and 48. High levels of emotional exhaustion and depersonalization as well as low levels of personal accomplishment are considered indicative of burnout. Participants respond to the 22 items on a 7-point scale ranging from never (0) to every day (6). The original study that focused on the development of the scale [11] reported satisfactory estimates of internal consistency (Cronbach’s alpha) ranging between 0.69 and 0.92 and also provided evidence of convergent and discriminant validity. A review of the reliability values reported in selected studies using the MBI in educational settings indicated that the reliability of each of these studies generally ranged between 0.50 and 0.90, with the reliability of the depersonalization subscale typically being consistently lower than that of the other two subscales [45]. A previous South African study confirmed the factor structure of the MBI and provided reliability estimates ranging from 0.71 to 0.89 [46]. Maslach and colleagues [47] suggested the following cut-off scores for EE (low ≤ 16, moderate 17−26, high ≥ 27); DP (low ≤ 6, moderate 7−12, high ≥ 13); and PA (low ≤ 31, moderate 32−38, high ≥ 39). While recognizing the cautionary note raised by Schaufeli and Van Dierendonck [48] about using cross-national cut-off scores in relation to the MBI, the cut-off scores are merely used in the results section for illustrative purposes.

The CES-D scale assesses depression and consists of 20 symptoms. Respondents are asked to indicate how often they experienced each of the symptoms during the past week on a 4-point scale that ranges from rarely or none of the time (0) to most or all of the time (3). Scores on the CES-D range between 0 and 60. The CES-D scale has demonstrated satisfactory internal consistency (0.85–0.90) and test-retest reliability (0.51–0.67). Validity has been established through patterns of correlations with clinical ratings of depression [32]. When used with a sample of South African students, satisfactory reliability coefficients (*α* and *ω* > 0.90) for the CES-D scale have also been reported [49].

The BHS assesses the degree to which individuals’ cognitive schemata are associated with pessimistic expectations. It contains 20 statements, and respondents are expected to indicate whether each statement is “true” or “false”. Example items include “I do not expect to get what I really want” and “My future seems dark to me”. Scores on the BHS range between 0 and 20 and higher scores indicate a greater degree of hopelessness. Internal consistency of 0.93 has been reported for the BHS, with a concurrent validity of 0.074 with clinical ratings of hopelessness and 0.60 with other scales of hopelessness [33]. The BHS has previously been used in South Africa [49], and an alpha coefficient of 0.86 was reported in that study.

The STAI-T is a 20-item measure of trait anxiety. Responses are scored on a 4-point scale that ranges from almost never (1) to almost always (4). Scores on the STAI-T range between 20 and 80. Example items include: “I worry too much over something that really doesn’t matter” and “I get in a state of tension or turmoil as I think over my recent concerns and interests.” The STAI-T has been used in a wide variety of contexts (e.g., Denmark, Lebanon, and China), and reliability values exceeding 0.85 have generally been reported [50,51,52,53]. In South Africa, a reliability coefficient (Cronbach’s alpha) of 0.90 was reported for this scale [54].

The SWLS is the most widely used measure of life satisfaction. It consists of five items that are scored on a 7-point scale that ranges from strongly agree (7) to strongly disagree (1). Higher scores reflect higher levels of satisfaction with life. Evidence of construct, convergent, and discriminant validity, as well as satisfactory estimates of internal consistency (*α* > 0.75) have been reported [55]. In South Africa, Pretorius and Padmanabhanunni used the classical test theory and Mokken and Rasch’s analyses to confirm the reliability, validity, and unidimensional nature of the SWLS [56].

### 2.3. Procedure

Google Forms was used to develop an electronic version of all the measuring instruments. Permission was obtained from Facebook administrators of groups of teachers to distribute the link during the period April to June 2021. In addition, online meetings were held with some provincial education departments to explain the purpose of the study and to invite officials to assist with the distribution of the electronic questionnaire.

### 2.4. Ethics

Ethical approval for the study was granted by the Humanities and Social Sciences Ethics Committee of the University of the Western Cape (ethics reference number: HS21/3/8). Participants completed the survey anonymously and provided informed consent. Participants were also provided with the authors’ contact details for psychological counseling support in the event that completing the survey resulted in some distress.

### 2.5. Data Analysis

The IBM SPSS Statistics for Windows (version 26; IBM Corp., Armonk, NY, USA) was used for all analyses, which included descriptive statistics, reliability measures (alpha and omega), and the intercorrelations between variables. Regression analyses with gender, age, perceived vulnerability to disease, fear of COVID-19, role ambiguity, and role conflict as predictors and the dimensions of burnout as dependent variables were undertaken to determine possible antecedents of burnout. Further regression analyses with the indices of psychological well-being as the dependent variable and the dimensions of burnout as the predictors were carried out to determine the possible consequences of burnout. We examined the distribution of all scores for normality and also visually inspected all scatterplots for linearity. The indices of skewness ranged between 0.5 and −0.5 for most of the variables, reflecting that these variables had a symmetrical distribution. The exceptions were role ambiguity (skewness = 0.88), depersonalization (skewness = 0.86), and hopelessness (skewness = 0.91), indicating that, in these instances, the distributions were moderately skewed. The scatterplots confirmed the linear relationships between the variables.

## 3. Results

The descriptive statistics, reliability values, and intercorrelations are reported in Table 2. In terms of intercorrelations, all the dimensions of burnout were correlated with the following presumed antecedents of burnout: perceived infectability (EE: r(353) = 0.27, *p* < 0.001; DP: r(353) = 0.22, *p* < 0.001; PA: r(353) = −0.15, *p* = 0.005) and role ambiguity (EE: r(353) = 0.29, *p* < 0.001; DP: r(353) = 0.25, *p* < 0.001; PA: r(353) = −0.51, *p* < 0.001). The relationship between these variables and the dimensions of burnout were positive for emotional exhaustion and depersonalization and negative for personal accomplishment. This indicates that higher levels of perceived infectability and role ambiguity are associated with higher levels of emotional exhaustion and depersonalization and lower levels of personal accomplishment.

In addition to these predictor variables that all the dimensions of burnout were related to, certain dimensions of burnout were differentially related to other predictor variables: emotional exhaustion and depersonalization were positively related to fear of COVID-19 (EE: r(353) = 0.25, *p* < 0.001; DP: r(353) = 0.23, *p* < 0.001) and role conflict (EE: r(353) = 0.39, *p* < 0.001; DP: r(353) = 0.37, *p* < 0.001), thus indicating that higher levels of emotional exhaustion and depersonalization are associated with higher levels of fear of COVID-19 and role conflict. Germ aversion was positively associated with emotional exhaustion (r(353) = 0.11, *p* = 0.042). Gender was negatively associated with emotional exhaustion (r(353) = −0.14, *p* = 0.024) and positively associated with personal accomplishment (r(353) = 0.11, *p* = 0.048), indicating that women reported higher levels of emotional exhaustion and lower levels of personal accomplishment. Finally, in terms of the presumed predictor variables, age was positively associated with personal accomplishment (r(353) = 0.17, *p* < 0.001), indicating that older respondents reported higher levels of personal accomplishment.

In terms of variables presumed to be psychological consequences of burnout, emotional exhaustion and depersonalization were positively related to depression (EE: r(353) = 0.53, *p* < 0.001; DP: r(353) = 0.41, *p* < 0.001), hopelessness (EE: r(353) = 0.49, *p* < 0.001; DP: r(353) = 0.36, *p* < 0.001), and anxiety (EE: r(353) = 0.57, *p* < 0.001; DP: r(353) = 0.39, *p* < 0.001), as well as negatively related to life satisfaction (EE: r(353) = −0.033, *p* < 0.001; DP: r(353) = −0.24, *p* < 0.001). Thus, high levels of emotional exhaustion and depersonalization were associated with higher levels of depression, hopelessness, and anxiety as well as lower levels of life satisfaction. Personal accomplishment, however, was negatively related to depression (r(353) = −0.48, *p* < 0.001), hopelessness (r(353) = −0.42, *p* < 0.001), and anxiety (r(353) = −0.42, *p* < 0.001) and positively related to life satisfaction (r(353) = 0.47, *p* < 0.001). This indicates that higher levels of emotional exhaustion and depersonalization, as well as lower levels of personal accomplishment, were associated with higher levels of depression, hopelessness, and anxiety and lower levels of life satisfaction.

The mean scores for the various dimensions of burnout were as follows: EE = 25.0 (±15.2), DP = 7.5 (±7.4), and PA = 32.0 (±11). A systematic review of 94 studies reported mean scores of 20.6 for emotional exhaustion, 6.6 for depersonalization, and 28.7 for personal accomplishment [57]. The mean scores in the current study were significantly higher with respect to emotional exhaustion (t(354) = 5.44, *p* < 0.001) and depersonalization (t(354) = 2.28, *p* = 0.023), but also higher in terms of personal accomplishment (t(354) = 5.63, *p* < 0.001) when compared to those reported in the systematic review. In terms of these cut-off scores, 43.4% and 21.1% reported high and moderate emotional exhaustion, respectively; additionally, 19.2% and 23.1% reported high and moderate depersonalization, respectively, while 44.5% and 17.2% reported low and moderate personal accomplishment.

With respect to the internal consistency of the measuring instruments, the questionnaires, with the exception of the GA subscale, generally demonstrated very satisfactory reliability coefficients (*α* and *ω* = 0.78 to 0.92). The one exception was the GA subscale, which had moderate but acceptable reliability (*α* = 0.65; *ω* = 0.66).

The results of the regression analysis with the dimensions of burnout as dependent variables and demographic variables (gender and age), perceived vulnerability to disease, fear of COVID-19, role conflict, and role ambiguity as independent variables are reported in Table 3. This table reveals that the variables presumed to be antecedents of burnout were all associated with one or more dimensions of burnout, with the exception of germ aversion.

The predictors of emotional exhaustion and depersonalization included role ambiguity (EE: *β* = 0.231, *p* < 0.001; DP: *β* = 0.257, *p* < 0.001), role conflict (EE: *β* = 0.341, *p* < 0.001; DP: *β* = 0.363, *p* < 0.001), and fear of COVID-19 (EE: *β* = 0.163, *p* < 0.001; DP: *β* = 0.141, *p* = 0.005). The predictors of personal accomplishment included role ambiguity (*β* = −0.457, *p* < 0.001) and perceived infectability (*β* = −0.105, *p* = 0.05). The only predictor of personal accomplishment and depersonalization was age (PA: *β* = 0.136, *p* = 0.003; DP: *β* = −0.114, *p* = 0.013). Finally, gender was a significant predictor of emotional exhaustion (*β* = −0.104, *p* = 0.026).

Predictors of psychological well-being on the basis of dimensions of burnout are reported in Table 4. All the dimensions of burnout were significant predictors of indices of psychological well-being, with the exception of depersonalization, which predicted life satisfaction.

Table 4 indicates that emotional exhaustion was a significant predictor of all indices of psychological well-being, including depression (*β* = 0.289, *p* < 0.001), hopelessness (*β* = 0.275, *p* < 0.001), anxiety (*β* = 0.378, *p* < 0.001), and life satisfaction (*β* = −0.132, *p* = 0.047). Similarly, personal accomplishment was a significant predictor of all indices, including depression (*β* = −0.334, *p* < 0.001), hopelessness (*β* = −0.290, *p* < 0.001), anxiety (*β* = −0.261, *p* < 0.001), and life satisfaction (*β* = 0.401, *p* < 0.001). Depersonalization predicted all the indices of psychological well-being (except life satisfaction) including depression (*β* = 0.168, *p* = 0.006), hopelessness (*β* = 0.143, *p* = 0.025), and anxiety (*β* = 0.130, *p* = 0.032).

## 4. Discussion

Burnout is a highly prevalent psychological syndrome among schoolteachers, and studies conducted during the pandemic have reported an escalation in symptoms of burnout among this population group [23]. Existing studies have confirmed that burnout is negatively associated with work engagement, job satisfaction, and physical and mental health outcomes [16]. In this study, we examined the potential role of demographic variables and COVID-19-related variables as predictors of the dimensions of burnout, as well as burnout as a predictor of psychological well-being. There were several important findings.

First, the demographic variables of gender and age were differentially related to the dimensions of burnout. Gender predicted emotional exhaustion, with women more likely to report emotional exhaustion. Existing studies have produced mixed results in terms of the role of gender in burnout. Some studies have found no gender differences in the experience of burnout [58,59], while others report inconsistent results in terms of the relationship between gender and the various dimensions of burnout. For example, Sak [60] found that men had higher emotional exhaustion and depersonalization scores than women, while men had lower scores for personal accomplishment than women. Nevertheless, other studies (e.g., [61]) have reported the same results as the current study—namely, that women report higher levels of emotional exhaustion than men (e.g., [62]). While our study is supported by a meta-analysis of 183 studies that found that women report higher levels of emotional exhaustion than men, Maslach and colleagues cautioned against a simplistic interpretation of gender differences with respect to burnout, arguing that gender differences might be the result of confounding gender with occupation (teachers are more likely to be women; soldiers are more likely to be men).

We also found that age predicted depersonalization and personal accomplishment, with older participants reporting lower levels of depersonalization and higher levels of personal accomplishment than younger participants. With respect to depersonalization, our findings contradict a meta-analysis of correlates of burnout that found that higher ages were associated with higher levels of depersonalization [63]; our findings also contradicted a study that found that older participants report higher levels of emotional exhaustion and depersonalization [64]. However, the latter study also found, in line with our results, that older participants report higher levels of personal accomplishment. This finding makes sense as older teachers are established for a longer period in their career and are, thus, likely to have accomplished more than younger teachers. However, an overview of the research conducted on burnout indicates that age differences in burnout experiences might just be an artifact of survival bias; those who experienced burnout early in their careers might have already dropped out of the profession, so it is possible that only those with low levels of burnout remain in the profession [65].

In terms of the COVID-19-related variables, fear of COVID-19 predicted emotional exhaustion and depersonalization, while perceived infectability predicted personal accomplishment. In a study of Filipino teachers during the COVID-19 pandemic, it was found that fear of COVID-19 was significantly associated with remote teaching burnout [66]. Similarly, in a sample of Egyptian physicians, it was found that fear of COVID-19 was significantly associated with all dimensions of burnout [67]; this is partly consistent with the results obtained in the current study. Experiencing fear of COVID-19 is a stressful state, and the relationship between stress and burnout is well-established in the research literature (e.g., [68,69]). It is likely that the same mechanism underlies perceived infectability; in other words, perceiving oneself as more susceptible to infections may lead to increased stress, in turn leading to burnout. In the South African context, it is likely that teachers experienced a heightened fear of COVID-19 owing to the resumption of traditional schooling and difficulties implementing personal protective measures in schools. Overcrowded classrooms, lack of access to clean running water, and limited personal protective equipment could have contributed to teachers fearing the possibility of contagion.

Role stress, in the form of role ambiguity and role conflict, was a significant predictor of all the dimensions of burnout with the exception of role conflict, which was not a significant predictor of personal accomplishment. These results are consistent with findings in other studies regarding the relationship between role stress and burnout (e.g., [70,71]). In the context of this pandemic, during which teachers rapidly had to adjust to new ways of working, the potential for role conflict and role ambiguity is significant. Being confused about the new parameters of one’s role (role ambiguity) and the irreconcilable demands of wanting to deliver effective teaching while doing so either remotely or on a rotational basis (role conflict) increases stress levels, which leads to burnout.

In terms of the relationship between burnout and indices of psychological well-being, all the dimensions of burnout significantly predicted depression, hopelessness, anxiety, and life satisfaction, with the exception of depersonalization, which was not a significant predictor of life satisfaction. These findings confirm the well-established association between burnout and psychological well-being (e.g., [72,73]).

The findings of this study have shown that multiple factors could lead to burnout, which is, in turn, associated with negative psychological well-being. As indicated, teaching is a highly stressful occupation with an increased risk of burnout. This necessitates investment in programs and interventions to help teachers cope with their stressful work environments. Maslach and colleagues [65] indicated that interventions aimed at burnout reduction have either focused on the individual or on the organization. At an individual level, this could include psycho-educational approaches or evidence-based therapies aimed at teaching people to cope with stress. At an organizational level, this could include support from school leadership, workplace wellness promotion programs, and mentoring programs. School leadership could also receive training to monitor the early warning signs of burnout and, thus, identify teachers at risk of burnout. Finally, authorities should do more to ensure that the working environment of teachers is conducive to quality learning and teaching.

The study has certain limitations. The cross-sectional nature of the data precludes the drawing of any causal inferences. Participation was voluntary, potentially leading to selection bias. Additionally, self-report measures, which are vulnerable to social desirability effects, were used. The majority of the sample were female and from one geographic area. In future studies, a more diverse sample could further corroborate our results.

## 5. Conclusions

Teachers are among a country’s most valuable resources. Teaching, however, is one of the most stressful occupations, and the mental health of teachers is often neglected. This could potentially lead to increased disillusionment with the teaching profession, high turnover, and negative student learning outcomes. To better understand burnout syndrome in teachers, this study examined multiple factors associated with burnout as well as the relationship between burnout and psychological well-being. We found that demographic variables, COVID-19-related variables, and role stress were significantly associated with the dimensions of burnout. The dimensions of burnout also predicted depression, hopelessness, anxiety, and life satisfaction. This study underscores the need for interventions to improve the working conditions of teachers and actively implement programs aimed at reducing burnout.

## Figures and Tables

**Table 1:** Presumed antecedents and consequences of burnout.

Antecedents	Burnout	Consequences
Gender	Emotional exhaustion	Depression
Age	Depersonalization	Hopelessness
Perceived vulnerability to disease	Personal accomplishment	Anxiety
Perceived infectability		Life satisfaction
Germ aversion		
Role stress		
Role ambiguity		
Role conflict		

**Table 2:** Descriptive statistics, reliability values, and intercorrelations between variables.

Variable	1	2	3	4	5	6	7	8	9	10	11	12	13	14
1. Gender	—													
2. Age	0.12 *	—												
3. PI	−0.02	0.09	—											
4. Germ Aversion	0.01	0.09	0.35 **	—										
5. Fear of COVID-19	−0.09	0.05	0.41 **	0.25 **	—									
6. Role Ambiguity	−0.05	−0.07	0.13 *	−0.24 *	0.04	—								
7. Role Conflict	0.06	0.03	0.21 **	0.18 **	0.12 *	0.04	—							
8. EE	−0.14 *	−0.08	0.27 **	0.11 *	0.25 **	0.29 **	0.39 **	—						
9. DP	−0.05	−0.10	0.22 **	0.06	0.23 **	0.24 **	0.37 **	0.61 **	—					
10. PA	0.11 *	0.17 **	−0.15 **	0.15 **	−0.09	−0.51 **	−0.04	−0.33 **	−0.29 **	—				
11. Depression	−0.11 *	−0.12 *	0.33 **	0.04	0.28 **	0.38 **	0.23 **	0.53 **	0.41 **	−0.48 **	—			
12. Hopelessness	−0.13 *	0.07	0.25 **	0.01	0.25 **	0.37 **	0.22 **	0.49 **	0.36 **	−0.42 **	0.61 **	—		
13. Anxiety	−0.17 **	−0.19 **	0.38 **	0.13 *	0.33 **	0.34 **	0.27 **	0.57 **	0.39 **	−0.42 **	0.74 **	0.62 **	—	
14. Life Satisfaction	0.08	0.07	−0.16 **	0.07	−0.11 *	−0.42 **	−0.09	−0.33 **	−0.24 **	0.47 **	−0.55	−0.62 **	−0.52 **	—
Mean	—	41.9	28.7	42.9	20.9	14.7	30.4	25.0	7.5	32.0	22.0	5.7	44.9	21.9
SD	—	12.4	8.8	8.4	7.1	5.7	8.2	15.2	7.4	11.0	12.2	4.9	10.3	7.3
Minimum	–	23	7	11	7	6	8	0	0	4.80	0	0	20	5
Maximum		73	49	56	35	36	48	42	42	48	57	20	73	35
Alpha	—	—	0.78	0.65	0.91	0.83	0.83	0.94	0.85	0.84	0.92	0.89	0.91	0.90
Omega	—	—	0.78	0.66	0.91	0.83	0.83	0.94	0.86	0.84	0.93	0.89	0.91	0.90

Note. PI = perceived infectability, EE = emotional exhaustion, DP = depersonalization, PA = personal accomplishment. ** *p* < 0.01, * *p* < 0.05.

**Table 3:** Predicting burnout with presumed antecedents.

Predictors	Beta	SE	Β	95% CI	*p*
Emotional Exhaustion (R^2^ = 0.277)					
Gender	−2.875	1.284	−0.104	[−0.540, −0.351]	0.026
Age	−0.062	0.045	−0.065	[−0.150, 0.025]	0.163
Germ Aversion	0.032	0.074	0.023	[−0.113, 0.178]	0.663
Perceived Infectability	0.123	0.074	0.090	[−0.022, 0.268]	0.096
Fear of COVID-19	0.273	0.085	0.163	[0.105, 0.441]	0.001
Role Ambiguity	0.489	0.103	0.231	[0.287, 0.692]	0.001
Role Conflict	0.497	0.069	0.341	[0.362, 0.632]	0.001
Depersonalization (R^2^ = 0.298)					
Gender	−1.852	1.08	−0.078	[−3.984, 0.280]	0.088
Age	−0.094	0.038	−0.114	[−0.169, −0.020]	0.013
Germ Aversion	0.036	0.062	0.030	[−0.086, 0.159]	0.561
Perceived Infectability	0.095	0.062	0.081	[−0.028, 0.217]	0.129
Fear of COVID-19	0.202	0.072	0.141	[0.061, 0.344]	0.005
Role Ambiguity	0.467	0.087	0.257	[0.296, 0.638]	0.001
Role Conflict	0.453	0.058	0.363	[0.339, 0.567]	0.001
Personal Accomplishment (R^2^ = 0.295)					
Gender	1.476	1.167	0.058	[−0.819, 3.771]	0.207
Age	0.120	0.041	0.136	[0.040, 0.200]	0.003
Germ Aversion	0.112	0.067	0.086	[−0.020, 0.244]	0.096
Perceived Infectability	−0.132	0.067	−0.105	[−0.264, 000]	0.050
Fear of COVID-19	−0.073	0.077	−0.047	[−0.225, 0.080]	0.348
Role Ambiguity	−0.890	0.094	−0.457	[−1.074, −0.706]	0.001
Role Conflict	−0.027	0.062	−0.020	[−0.150, 0.096]	0.665

**Table 4:** Dimensions of burnout as predictors of indices of psychological well-being.

Predictors	Beta	SE	Β	95% CI	*p*
Depression (R^2^ = 0.389)					
Emotional Exhaustion	0.295	0.061	0.289	[0.175, 0.414]	0.001
Depersonalization	0.200	0.072	0.168	[0.059, 0.341]	0.006
Personal Accomplishment	−0.371	0.049	−0.334	[−0.468, −0.273]	0.001
Hopelessness (R^2^ = 0.314)					
Emotional Exhaustion	0.113	0.026	0.275	[0.062, 0.165]	0.001
Depersonalization	0.069	0.031	0.143	[0.009, 0.129]	0.025
Personal Accomplishment	−0.130	0.021	−0.290	[−0.172, −0.089]	0.001
Anxiety (R^2^ = 0.382)					
Emotional Exhaustion	0.325	0.052	0.378	[0.224, 0.427]	0.001
Depersonalization	0.130	0.061	0.130	[0.011, 0.250]	0.032
Personal Accomplishment	−0.245	0.042	−0.261	[−0.327, −0.162]	0.001
Life Satisfaction (R^2^ = 0.251)					
Emotional Exhaustion	−0.080	0.040	−0.132	[−0.159, −0.001]	0.047
Depersonalization	−0.053	0.047	−0.075	[−0.146, 0.040]	0.260
Personal Accomplishment	0.266	0.033	0.401	[0.201, 0.330]	0.001

## Data Availability

The raw data supporting the conclusions of this article will be made available by the authors, without undue reservation.
